# Integrin Beta 4E Promotes Endothelial Phenotypic Changes and Attenuates Lung Endothelial Cell Inflammatory Responses

**DOI:** 10.3389/fphys.2022.769325

**Published:** 2022-02-18

**Authors:** Weiguo Chen, Jamie M. C. Gard, Yulia Epshtein, Sara M. Camp, Joe G. N. Garcia, Jeffrey R. Jacobson, Anne E. Cress

**Affiliations:** ^1^Department of Medicine, Division of Pulmonary, Critical Care, Sleep and Allergy, University of Illinois at Chicago, Chicago, IL, United States; ^2^Department of Cellular and Molecular Medicine, University of Arizona, Tucson, AZ, United States; ^3^Department of Medicine, University of Arizona Health Sciences, Tucson, AZ, United States

**Keywords:** integrins, inflammation, acute lung injury, lung endothelial cells, permeability

## Abstract

We previously reported integrin beta 4 (ITGB4) is an important mediator of lung vascular protection by simvastatin, a 3-hydroxy-3-methylglutaryl-coenzyme A-reductase inhibitor. In this study, we report increased endothelial cell (EC) expression specifically of ITGB4E, an ITGB4 mRNA splice variant, by simvastatin with effects on EC protein expression and inflammatory responses. In initial experiments, human pulmonary artery ECs were treated using simvastatin (5 μM, 24 h) prior to immunoprecipitation of integrin alpha 6 (ITGA6), which associates with ITGB4, and Western blotting for full-length ITGB4 and ITGB4E, uniquely characterized by a truncated 114 amino acid cytoplasmic domain. These experiments confirmed a significant increase in both full-length ITGB4 and ITGB4E. To investigate the effects of increased ITGB4E expression alone, ECs were transfected with ITGB4E or control vector, and cells were seeded in wells containing Matrigel to assess effects on angiogenesis or used for scratch assay to assess migration. Decreased angiogenesis and migration were observed in ITGB4E transfected ECs compared with controls. In separate experiments, PCR and Western blots from transfected cells demonstrated significant changes in EC protein expression associated with increased ITGB4E, including marked decreases in platelet endothelial cell adhesion molecule-1 (PECAM-1) and vascular endothelial-cadherin (VE-cadherin) as well as increased expression of E-cadherin and N-cadherin along with increased expression of the Slug and Snail transcription factors that promote endothelial-to-mesenchymal transition (EndMT). We, then, investigated the functional effects of ITGB4E overexpression on EC inflammatory responses and observed a significant attenuation of lipopolysaccharide (LPS)-induced mitogen-activated protein kinase (MAPK) activation, including decreased phosphorylation of both extracellular signal-regulated kinase (ERK) and c-Jun N-terminal kinase (JNK), as well as reduced inflammatory cytokines (IL-6 and IL-8), expressed in the media of EC after either LPS or excessive cyclic stretch (CS). Finally, EC expression-increased ITGB4E demonstrated decreased barrier disruption induced by thrombin as measured by transendothelial electrical resistance. Our data support distinct EC phenotypic changes induced by ITGB4E that are also associated with an attenuation of cellular inflammatory responses. These findings implicate ITGB4E upregulation as an important mediator of lung EC protection by statins and may lead to novel therapeutic strategies for patients with or at risk for acute lung injury (ALI).

## Introduction

The statin drugs are a class of 3-hydroxy-3-methylglutaryl-coenzyme A-reductase inhibitors (HMG CoA-reductase inhibitors) most commonly used clinically to lower serum cholesterol levels and for their protective effects with respect to cardiovascular disease. However, these drugs are also recognized to have pleiotropic properties (Davignon, 2004; Jacobson, 2009; Zhang et al., 2020). We previously reported lung vascular-protective effects of simvastatin in acute inflammatory lung injury models with protection mediated by simvastatin-mediated upregulation of integrin beta 4 (ITGB4), a key transmembrane protein, in lung endothelial cells (ECs) (Jacobson et al., 2005; Chen et al., 2012). The role of ITGB4 in acute inflammatory lung injury models, however, is complex as the attenuation of lung EC inflammatory responses by simvastatin is associated with increased expression of ITGB4 but decreased ITGB4 tyrosine phosphorylation.

There are 8 separate integrin beta subunits with the laminin-5 receptor ITGB4, which forms a heterodimer only with integrin α6, uniquely characterized by an extended cytoplasmic tail (>1,000 amino acids). This tail is comprised of a proximal Calx Na-Ca exchanger domain followed by two pairs of fibronectin type III repeats separated by a tyrosine activation motif (TAM) (Hogervorst et al., 1990). The extracellular portion of the ITGB4 subunit sequence has 35% of homology with other integrin beta subunits but is the most distinct within this class of molecules, and the cytoplasmic domain shows no substantial homology to the cytoplasmic tails of the other beta sequences. The exceptionally long cytoplasmic domain of ITGB4 suggests potentially distinct protein interactions and signaling. ITGB4 has been identified as a mediator of mitogen-activated protein kinase (MAPK) signaling in a variety of cell types (Nikolopoulos et al., 2005; Meng et al., 2020), and we previously reported that ITGB4 regulates lung EC inflammatory responses *via* effects on MAPK signaling mediated by SHP-2, a protein tyrosine phosphatase (Chen et al., 2010). Subsequent studies identified variable effects on EC inflammatory responses associated with the overexpression of ITGB4 mutants characterized by specific mutations or deletions within the regions of the cytoplasmic tail (Chen et al., 2015).

In addition to increased EC expression of full-length ITGB4 after simvastatin treatment, we recently identified increased expression of ITGB4E, a unique ITGB4 mRNA splice variant. ITGB4E lacks several specific domains found in the canonical ITGB4 cytoplasmic region and contains a unique cytoplasmic sequence of 114 amino acid residues (Kelly et al., 2020). In this study, we sought to characterize specifically the effects of ITGB4E overexpression on lung EC phenotypic changes and to explore the role of ITGB4E as a modulator of EC inflammatory responses.

## Materials and Methods

### Antibodies and Reagents

ITGB4 antibody was purchased from Santa Cruz Biotechnology (Santa Cruz, CA, United States). The Lentivirus ELISA kit was purchased from ZeptoMetrix (Buffalo, NY, United States). A protease and phosphatase inhibitor cocktail was purchased from Calbiochem (San Diego, CA, United States), and a bicinchoninic acid protein assay kit was purchased from Pierce (Rockford, IL, United States). ECM Gel from Engelbreth-Holm-Swarm murine sarcoma (Matrigel) was obtained from MilliporeSigma (Burlington, VT, United States). Unless otherwise stated, all other antibodies and reagents were purchased from Cell Signaling Technology (Beverly, MA, United States).

### Endothelial Cell Culture

Human pulmonary artery ECs from Lonza (Walkersville, MD, United States) were cultured in an essential growth medium (EGM-2) containing 10% of fetal bovine serum (Lonza). Cells were placed in an incubator at 37°C, 5% of CO_2_, and 95% of humidity to achieve contact-inhibited monolayers.

### ITGB4E Overexpression in Endothelial Cell

The ITGB4E variant DNA sequence was subcloned into a pLV-eGFP lentiviral mammalian expression vector (VectorBuilder, Chicago, IL, United States) (see supplementary material). Control vector (pLV-eGFP) or pLV carrying ITGB4E was then co-transfected into 293NT cells with packaging plasmid psPAX2, a gift from Didier Trono (Addgene plasmid #12260;^1^ RRID:Addgene_12260) and envelope plasmid pMD2.g, a gift from Didier Trono (Addgene plasmid #12259;^2^ RRID:Addgene_12259).

Briefly, 12 μg of psPAX2 plasmid, 5 μg of pMD2.G plasmid, and 15 μg of ITGB4E or vector plasmid DNA were added to 80 μl of Lipofectamine 2000 in 3.5 ml of Opti-MEM for 10 min at room temperature and then applied to a 15-cm plate of 293NT cells. The medium (Opti-MEM) was changed on the following day. On day 3, the medium was collected and centrifuged at 26,000 rpm for 90 min. The pellets were resuspended in phosphate-buffered saline (PBS), and the virus concentration was measured using an HIV-1 p24 antigen ELISA kit (ZeptoMetrix, NY, United States). The virus was then incubated with polybrene transfection reagent (TR-1003-G, MilliporeSigma, St. Louis, MO, United States) for 10 min at room temperature and then applied to human pulmonary artery ECs in media. The expression efficiency was determined by green fluorescent protein (GFP) detectable using fluorescent microscopy, and GFP-positive cells were then sorted using flow cytometry (Moflo Astrios Flow Cytometry Cell Sorter, Beckman Coulter, Indianapolis, IN, United States). Finally, purified cells were cultured in EGM in the presence of 1 μg/ml of puromycin for 2 days after which cells were used for experiments as described.

### Immunoblotting

Total proteins were extracted using NP-40 lysis buffer (50 mM of Tris–HCl, pH 7.4, 150 mM of NaCl, 1% of NP-40, and 5 mM of ethylenediaminetetraacetic acid) supplemented with 40 mM of sodium fluoride, 0.1 M of sodium orthovanadate, 0.2 mM of phenylmethylsulfonyl fluoride, 10 mM of *N*′ ethylmalemide, and protease and phosphatase inhibitor cocktail (Calbiochem). Lung homogenates and cell lysates were briefly sonicated and were subjected to cycles of thawing and freezing on dry ice. The protein concentrations were measured using a bicinchoninic acid protein assay kit (Pierce). Western blotting was performed using standard protocols, and band densities were determined using ImageJ software (National Institutes of Health^3^).

### Angiogenesis Assay

Angiogenesis was assessed as we have described previously (Linz-Mcgillem et al., 2004). Briefly, 1 × 10^5^ ECs were seeded into 12-well plates containing Matrigel in EGM-2. On day 1 after seeding, representative images were obtained using fluorescence microscopy (Olympus BX 51/IX70, Olympus, Japan). Total segment lengths relative to the number of anchorage joints were quantified using ImageJ software as described by others (Carpentier et al., 2020).

### Scratch-Wound Assay

To assess migration, 2 × 10^5^ ECs were seeded into 6-well plates in EGM-2 that were changed on day 2. Cells were then scratched using a 200 μl pipette tip, washed with PBS, and then placed in EGM-2. Images were obtained using fluorescence microscopy (Olympus BX 51/IX70, Olympus, Japan). For quantification of gap closure, the gap area after the scratch was measured at the time of scratch and again on day 1 using ImageJ software as described by others (Suarez-Arnedo et al., 2020).

### Measurement of Inflammatory Cytokine Expression After Lipopolysaccharide or Cyclic Stretch

For the LPS challenge, ECs were cultured in EGM-2 and challenged with 100 ng/ml of LPS for various time points as indicated. The resulting media were collected and briefly centrifuged using ELISA assay (Biolegend, San Diego, CA, United States) according to the manufacturer’s instructions. For CS experiments, ECs were plated onto six-well silicone elastomer Bioflex plates coated with type I collagen (FlexCell International, Hillsborough, NC, United States) and grown to 75–80% prior to transfection as described. Mechanical stretch was performed *via* the Flexcell Strain Unit (FX-3000; FlexCell International) placed in a 5% of CO_2_ incubator at 37°C and 95% of humidity. The device uses a controlled vacuum to induce CS with 18% of elongation at a frequency of 30 cycles per minute (0.5 Hz) for 4 h. The media were then collected and briefly centrifuged prior to the ELISA assay.

### Measurement of Transendothelial Electrical Resistance

Endothelial cells were plated into polycarbonate wells containing evaporated gold microelectrodes to measure TER that evaluates real-time changes in cell morphology, attachment, and locomotion using an electric cell-substrate impedance system (ECIS) (Applied Biophysics, Troy, NY, United States) as previously described (Garcia et al., 2001). Cells were grown to confluence in EGM-2. EC monolayers were then treated with thrombin (1 U/ml) to induce barrier disruption. TER values from each microelectrode were pooled at discrete time points and plotted vs. time as the mean ± SEM.

### Statistics

The Shapiro–Wilk’s test for normality was used. Student’s *t*-test was used to compare the means of data from two experimental groups while significant differences (*p* < 0.05) among multiple group comparisons were confirmed by a one-way ANOVA followed by Tukey’s range test. Results are expressed as means ± SE.

## Results

### Effect of Simvastatin on Lung Endothelial Cell ITGB4E Expression

**FIGURE 1:**
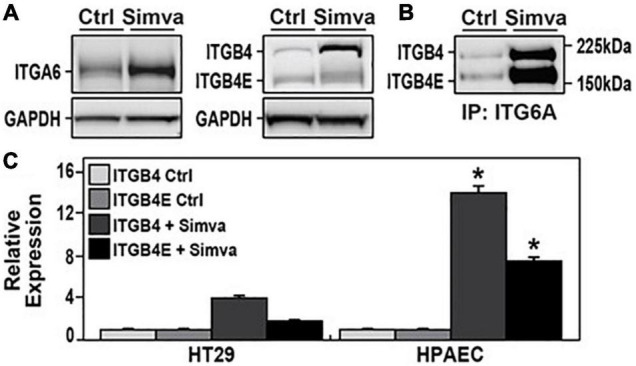
Increased expression of integrin beta 4 (ITGB4) and integrin beta 4E (ITGB4E) in human lung endothelial cells (ECs) after simvastatin. **(A)** Human pulmonary artery ECs were treated with simvastatin (5 μM, 24 h) and lysates were collected and subjected to Western blotting for ITGA6 and ITGB4. **(B)** In separate experiments, lysates from simvastatin-treated ECs were used for immunoprecipitation of ITGA6 followed by Western blotting for ITGB4. **(C)** Human colon adenocarcinoma cells (HT-29) and human pulmonary artery ECs were treated with simvastatin (5 mM, 24 h) and lysates were subjected to Western blotting for ITGB4. Relative densitometry is shown (*n* = 3 independent experiments, *p < 0.05 compared with both untreated ECs and simvastatin-treated HT-29 cells).

In the initial experiments, human pulmonary artery ECs were treated with simvastatin (5 μM, 24 h), and lysates were collected for Western blotting for ITGA6 and ITGB4 (Figure 1). These experiments confirmed a significant increase in expression in both proteins induced by simvastatin with two distinct bands observed after probing for ITGB4. The 202 kDa immunoreactive band is consistent with full-length ITGB4, and the second band at ∼150 kDa is consistent with ITGB4E. Furthermore, immunoprecipitation of ITGA6 followed by Western blotting for ITGB4 confirmed increased heterodimer formation of both ITGB4 and ITGB4E with ITGA6 after simvastatin treatment. These experiments were repeated to compare cell-specific responses using human pulmonary artery ECs and human colon adenocarcinoma cells (HT-29). ITGB4 and ITGB4E were both significantly increased in simvastatin-treated ECs relative to simvastatin-treated HT-29 cells and untreated ECs.

### Overexpression of ITGB4E in Lung Endothelial Cells

**FIGURE 2:**
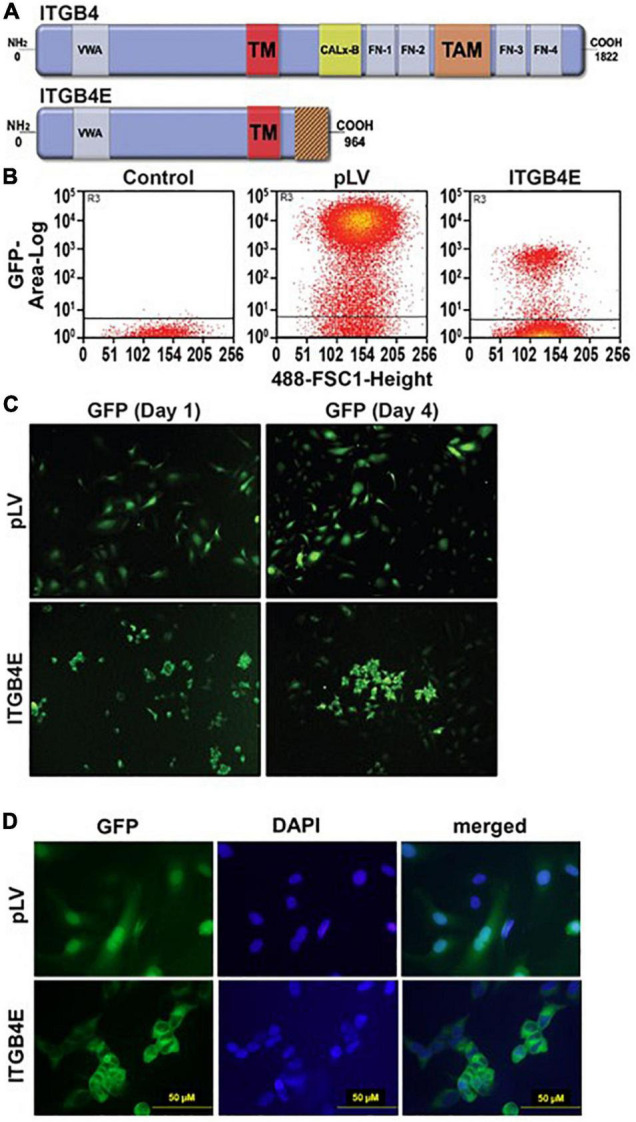
Overexpression of ITBG4E in human lung ECs. **(A)** ITGB4E structure. While full-length ITGB4 is characterized by a 1,000 amino acid cytoplasmic tail, ITGB4E has a unique, truncated 114 amino acid cytoplasmic domain (TM, transmembrane domain; PSI, plexin-semaphorin-integrin domain; VWA, von Willebrand factor A domain; TM, transmembrane domain; FN, fibronectin type III domain; TAM, tyrosine activation motif). **(B)** Human pulmonary artery ECs were transfected with ITBG4E-GFP or control vector (pLV-GFP) with green fluorescent protein (GFP)-positive cells sorted using flow cytometry. **(C)** Transfected cells were cultured and imaged on days 0 and 4. **(D)** Separately, 4′,6-diamidino-2-phenylindole (DAPI) staining on day 4 was performed as well (representative images shown).

To study the effects of ITGB4E on EC expression and function, an ITBG4 construct was subcloned into pLV plasmid and then co-transfected with sPAX2 and pMD2.G into 293NT cells. The medium was collected, and the virus was then purified and transfected into human pulmonary artery ECs. GFP-positive cells were sorted using flow cytometry and cultured in EMG2. Images obtained on day 4 after transfection were notable for aggregation of cells overexpressing ITGB4E compared with cells transfected with vector alone (Figure 2). In addition, 4′,6-diamidino-2-phenylindole (DAPI) was used as a nuclear stain to assess cell morphology. Notably, these studies did not demonstrate changes consistent with significant apoptosis associated with ITGB4E overexpression.

### Functional Effect of ITGB4E Overexpression in Lung Endothelial Cells

**FIGURE 3:**
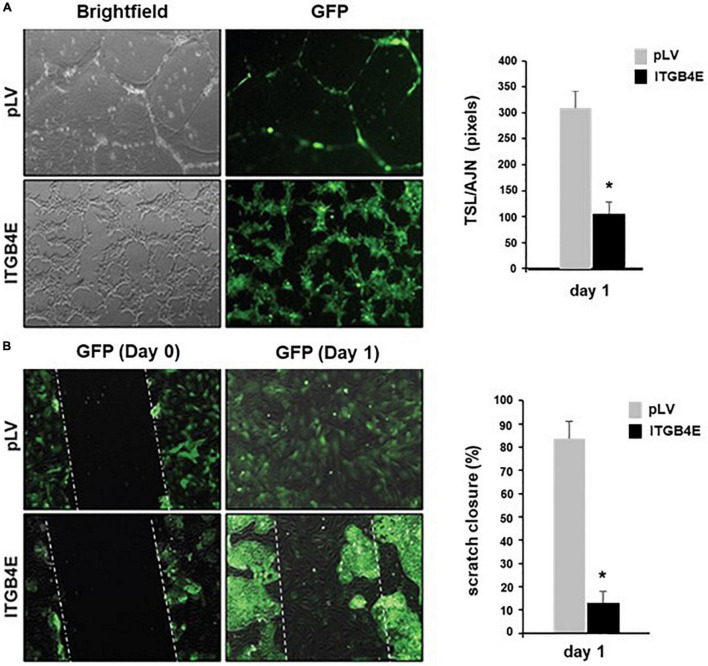
ITGB4E overexpression is associated with dysregulated EC angiogenesis and migration. **(A)** Human pulmonary artery ECs were transfected with ITGB4E or control vector and then grown in Matrigel. Images were then taken on day 1 to assess changes associated with angiogenesis (representative images shown). Quantitation of endothelial tube formation was performed and expressed as total segment length (TSL) relative to the number of anchorage joints (AJN) (*p < 0.01, *n* = 3 independent experiments). **(B)** In separate experiments, transfected cells were grown to confluence and used for scratch assay to assess migration. Images of cells overexpressing ITGB4E and controls were taken on days 0 and 1 (representative changes shown). Quantitation of relative gap closure on day 1 was performed and expressed as a percentage of the initial gap width on day 0 (*p < 0.01, *n* = 3 independent experiments).

Human pulmonary artery ECs were used for two separate assays to assess the effects of ITGB4 overexpression on angiogenesis and migration. To assess effects on angiogenesis, cells transfected with ITGB4E or control plasmids were grown in Matrigel, and images were obtained on day 1 after seeding (Figure 3A). Compared with control cells, characteristic changes associated with angiogenesis including cell elongation, tube formation, and branching were significantly diminished in cells overexpressing ITGB4E. To study the effects on stimulated migration, cells transfected with ITGB4E or control plasmids were grown to confluence in media prior to being scratched with a pipette tip to create a gap in the monolayer. Images were then taken on day 0 and then day 1 to assess relative gap closure (Figure 3B). While the initial gap created on day 0 was similar to both cell types, gap closure was complete with control cells on day 1 while cells transfected with ITGB4E demonstrated cell aggregation and little evidence of migration to achieve gap closure.

### Epithelial-Mesenchymal Transition Induced by ITGB4E Overexpression in Lung Endothelial Cells

**FIGURE 4:**
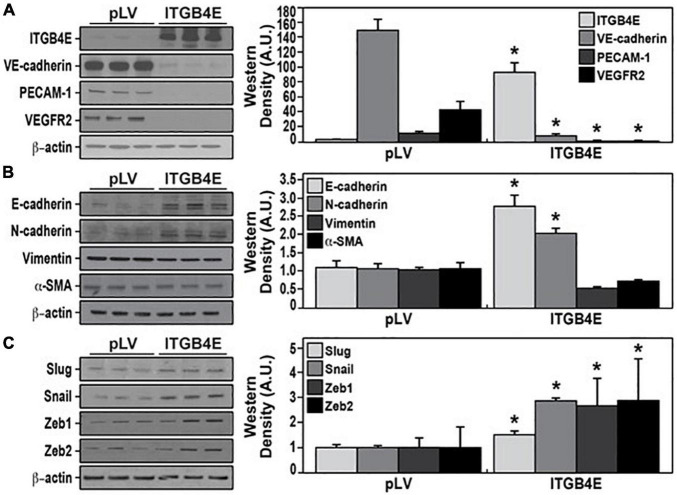
Overexpression of ITGB4E promotes endothelial-to-mesenchymal transition (EndMT). Human pulmonary artery ECs were transfected with ITGB4E or a control vector and lysates were then collected and subjected to Western blotting for **(A)** EC markers including VE-cadherin, PECAM-1, and VGFR2; **(B)** mesenchymal transition markers including E-cadherin, N-cadherin, vimentin, and alpha-smooth muscle actin (α-SMA); and **(C)** transcription factors known to mediate EndMT including Snail, Slug, Zeb-1, and Zeb-2 (*n* = 3 independent experiments, *p < 0.05 compared with respective controls).

Evidence of dysregulated EC angiogenesis and migration associated with overexpression of ITGB4E is consistent with a phenotypic change in these cells. To further characterize these changes, Western blots were performed using lysates from ITGB4E overexpressing and control cells probed for various EC marker proteins. These experiments confirmed nearly complete abrogation of the expression of VE-cadherin, PECAM-1, and VEGFR2 (Figure 4A). Additional experiments demonstrated significant increases in the expression of both E-cadherin and N-cadherin without altered expression of either vimentin or alpha-smooth muscle actin (α-SMA) (Figure 4B). As the ITGB4-mediated loss of EC markers in conjunction with increased N-cadherin is consistent with EMT, we further characterized potential EMT phenotypic changes. Western blots of ITGB4-overexpressing cells identified significant increases in the expression of Slug, Snail, Zeb-1, and Zeb-2 specific transcription factors known to mediate the spectrum of EMT (Figure 4C; Cano et al., 2000; Comijn et al., 2001; Bolos et al., 2003; Eger et al., 2005; Ma et al., 2021).

### Effect of ITGB4E Overexpression on Endothelial Cell Inflammatory Responses: MAPK Signaling

**FIGURE 5:**
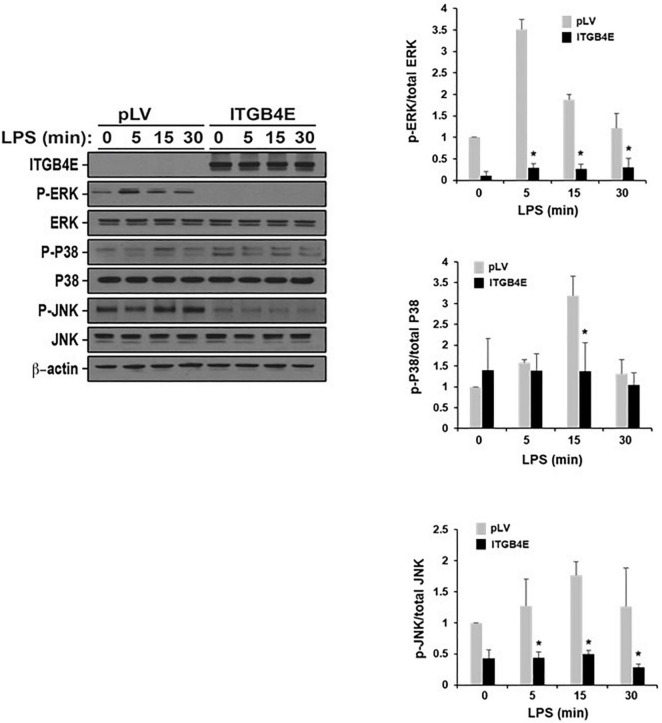
Reduced lipopolysaccharide (LPS)-induced EC mitogen-activated protein kinase (MAPK) activation associated with ITGB4E overexpression. Human pulmonary artery ECs were transfected with ITGB4E or control vector (pLV) prior to treatment with LPS (100 ng/ml) for various time points (0, 5, 15, or 30 min). Lysates were then collected and used for Western blotting for total and phosphorylated Erk, p38, and JNK (representative blots shown) (*p < 0.05 compared with pLV controls).

To investigate the effects of ITGB4E on EC signaling in response to inflammatory stimuli, human pulmonary artery ECs were transfected with ITGB4E or control vectors prior to treatment with LPS (100 ng/ml) for various time points (0–30 min). Lysates were then collected and subjected to Western blotting for specific MAPKs (Figure 5). These experiments confirmed a significant and consistent attenuation of LPS-induced Erk and JNK phosphorylation while no changes were appreciable with respect to total Erk and JNK. Of note, no changes were appreciable with respect to phosphorylated or total p38 MAPK. Collectively, these data are consistent with significant attenuation of EC inflammatory signaling by increased ITGB4E expression.

### Effect of ITGB4E Overexpression on Endothelial Cell Inflammatory Responses: Cytokine Expression

**FIGURE 6:**
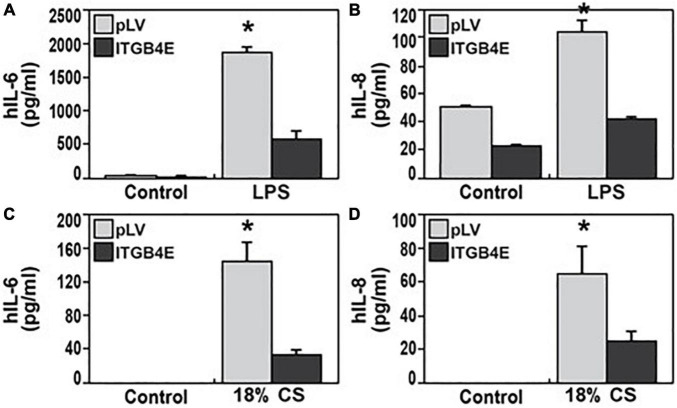
Reduced EC inflammatory responses associated with ITGB4E overexpression. Human pulmonary artery ECs were transfected with ITGB4E or control vector and then subjected to inflammatory stimuli, either LPS 100 ng/ml (4 h) or excessive cyclic stretch (CS, 18% elongation, 4 h). Media were then collected and used for measurements of inflammatory cytokines, human IL-6, and IL-8 [**(A,B)**, respectively, after LPS; **(C,D)**, respectively, after CS] (*n* = 3 independent experiments, *p < 0.05 compared with respective controls).

Next, in complementary experiments, human pulmonary artery ECs were transfected with ITGB4E or control vectors prior to being subject to distinct inflammatory stimuli, LPS (100 ng/ml, 4 h), or excessive mechanical stretch (18% CS, 4 h). Media were then collected and used for the measurement of inflammatory cytokines, IL-6 and IL-8, *via* ELISA (Figure 6). Increased ITGB4E expression was associated with a significant reduction of 75–80% in both IL-6 and IL-8 expressions in the media after either stimulus consistent with a decreased inflammatory response.

### Effect of ITGB4E on Lung Endothelial Cell Barrier Function

**FIGURE 7:**
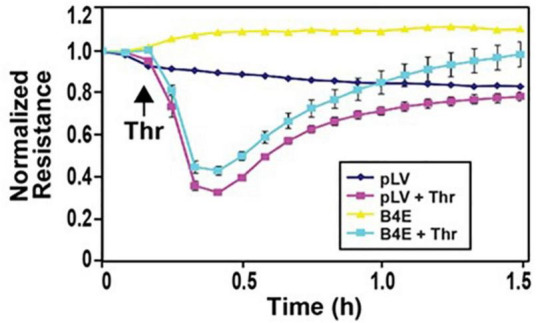
ITGB4E overexpression attenuates thrombin-induced EC barrier disruption. Human pulmonary artery ECs were transfected with ITGB4E or control vector and then plated on polycarbonate wells containing evaporated gold microelectrodes followed by treatment with thrombin (1 U/ml), a barrier- disruptive agonist, or vehicle and TER was measured. Thrombin-induced barrier disruption was attenuated in ITGB4E-overexpressing cells as evidenced by a greater nadir and a slower time to recovery in thrombin-treated control ECs (*n* = 3 independent experiments, *p* < 0.05 for ITGB4E-transfected cells compared with controls at each time point post-thrombin).

To determine the effect of ITGB4E overexpression on EC barrier function, human pulmonary artery ECs were transfected with ITBG4E or a control vector and then grown to confluence overlying gold-plated microelectrodes. Cells were then treated with thrombin (1 U/ml) to effect barrier disruption, and TER was measured (Figure 7). TER nadir and time to recovery after the treatment with thrombin (1 U/ml), a barrier-disrupting agent, were significantly decreased in cells overexpressing ITGB4E compared with controls. These changes are consistent with relative barrier protection associated with ITGB4E expression.

## Discussion

We have previously identified ITGB4 as an important mediator of the lung vascular-protective effects of statins observed in various inflammatory lung injury models. This study extends this work as we have now identified simvastatin-induced ITGB4E expression, an ITGB4 splice variant, attenuates EC inflammatory responses, a novel mechanism for the vascular protective effects of statins. Furthermore, our results are consistent with significant EC phenotypic changes associated with ITGB4E expression.

Integrin beta 4 is a transmembrane protein that has been identified as a prominent component of hemidesmosomes in epithelial cells (Stepp et al., 1990; Kurpakus et al., 1991). However, a large and growing body of literature has characterized the complex role of ITGB4 in cancer biology. For example, ITGB4 promotes cell invasion in hepatocellular carcinoma (Li et al., 2017) and breast cancer metastases (Abdel-Ghany et al., 2001), and ITGB4 expression has been identified as a prognostic marker in pancreatic ductal cancer, colon cancer, and head and neck squamous cell carcinomas (Nagata et al., 2013; Masugi et al., 2015; Li et al., 2019, 2020). Our recent study identified differential effects of ITGB4 splice variants on the migration of esophageal squamous cells and found ITGB4E is uniquely associated with decreased cancer cell migration (Kelly et al., 2020). Separately, we have characterized the role of ITGB4 as a mediator of lung vascular permeability and inflammatory responses in acute lung injury (ALI) models. To bring together these two distinct areas of investigation, we sought to define the effects of ITGB4E on lung EC signaling and function relevant to acute inflammatory lung injury. Our results confirm significantly attenuated inflammatory responses in EC overexpressing ITGB4E characterized by decreases in agonist-induced MAPK activation, expression of inflammatory cytokines, and EC barrier disruption. These effects were also associated with notable phenotypic changes including decreased expression of specific EC markers and increased expression of mesenchymal markers as well as transcription factors known to promote EndMT. Of note, while our experiments relied on the use of lung macrovascular ECs, our lab has previously reported that human pulmonary artery ECs have qualitatively similar responses to those of human lung microvascular ECs in the models employed (Mitra et al., 2021).

Endothelial-to-mesenchymal transition is defined by the phenotypic transition of ECs to that of mesenchymal cells and is characteristically associated with a loss of EC markers with increased expression of N-cadherin but also vimentin and α-SMA (Zeisberg et al., 2007; Xu-Dubois et al., 2016). This process is similar to EMT, which is characterized by the loss of epithelial markers including E-cadherin (Thiery, 2002; Eastham et al., 2007). While we found EC overexpression of ITGB4E promotes changes consistent with those seen in EndMT, several changes suggest a more complex transition, including increased expression of E-cadherin and no changes with respect to vimentin or α-SMA expression. Importantly, the fact that we did not observe changes with respect to vimentin and α-SMA indicates phenotypic changes that are distinct from those that fully define EndMT. Furthermore, evidence of increases in the transcription factors Snail, Slug, and Zeb 1/2 in ECs has also been described in cancer biology with effects on tumor growth, angiogenesis, and gene expression unrelated to EndMT (Hultgren et al., 2020; Rong et al., 2020; Cabrerizo-Granados et al., 2021).

Epithelial-mesenchymal transition and EndMT are now appreciated as a spectrum of states, which has complexity and mixed phenotypic characteristics resulting in response to environmental stimuli (Voon et al., 2017; Pal et al., 2021). In this regard, the appearance of the ITGB4E variant is particularly intriguing since as a product of mRNA splicing, the resulting unique mRNA may serve as an early marker for ALI and/or barrier repair. Alternatively, single nucleotide polymorphisms (SNPs) may influence the production of the splice variant since point mutations in esophageal cancers will influence ITGB4E production (Kelly et al., 2020), and others have reported nucleotide substitutions in ITGB4 far from the splice site result in a splicing abnormality that underlies the disease state of pyloric atresia-junctional epidermolysis bullosa syndrome (Masunaga et al., 2015). It would be of interest to determine whether SNPs within ITGB4 are associated with altered EC barrier function.

Our findings now further elucidate the complicated effects of statins on ITGB4 regulation. For example, while simvastatin upregulates ITGB4 expression, pretreatment with an ITGB4 blocking antibody attenuates simvastatin effects *in vitro* and *in vivo*, affecting increased ITGB4 phosphorylation and exacerbating ALI (Chen et al., 2012). A more precise explanation of the effects of statins on ITGB4 regulation should also acknowledge effects on both ITGB4E specifically, which we have now observed to have vascular-protective properties, and full-length ITGB4, which has variable effects on lung vascular inflammatory responses in the context of ALI.

Dysregulated migration of lung EC expressing the A6B4E heterodimer coupled with decreased agonist-induced barrier dysfunction as measured by TER suggests a unique role for the integrin variant, both as an early responder and as a mediator of adhesion. Notably, the unique cytoplasmic domain of the integrin variant contains amino acid sequences that would be predicted to bind integrin adapters of the kindlin-2 family (Rognoni et al., 2016; Bottcher et al., 2017). It remains to be determined whether kindlin-2 can, in fact, interact with the unique ITGB4E integrin cytoplasmic domain in addition to the known binding activities of the kindlin family for the B1 integrin to recruit paxillin to focal adhesions. Future work will be to determine whether ALI induction of ITGB4E expression will result in the recruitment of kindlin-2 and the subsequent assembly of paxillin-containing focal adhesions.

## Data Availability Statement

The raw data supporting the conclusions of this article will be made available by the authors, without undue reservation.

## Author Contributions

WC, AC, and JJ: experimental design and data interpretation. WC, YE, and JMG: experimental conduct. WC, SC, JGG, JJ, and AC: manuscript preparation. All authors have read and approved the final manuscript.

## Conflict of Interest

The authors declare that the research was conducted in the absence of any commercial or financial relationships that could be construed as a potential conflict of interest.

## Publisher’s Note

All claims expressed in this article are solely those of the authors and do not necessarily represent those of their affiliated organizations, or those of the publisher, the editors and the reviewers. Any product that may be evaluated in this article, or claim that may be made by its manufacturer, is not guaranteed or endorsed by the publisher.

## Footnotes

1
http://www.addgene.org/12260

2
http://www.addgene.org/12259

3
http://imagej.nih.gov/ij/

## Abbreviations

ALI: acute lung injury
CS: cyclic stretch
EC: endothelial cell
EMT: epithelial-mesenchymal transition
EndMT: endothelial-to-mesenchymal transition
ITGA6: integrin alpha 6
ITGB4/ITGB4E: integrin beta 4/integrin beta 4E
LPS: lipopolysaccharide
MAPK: mitogen-activated protein kinase
TAM: tyrosine activation motif
TER: transendothelial electrical resistance.

## References

[B1] Abdel-Ghany M. Cheng H. C. Elble R. C. Pauli B. U. (2001). The breast cancer beta 4 integrin and endothelial human CLCA2 mediate lung metastasis. *J. Biol. Chem.* 276 25438–25446. 10.1074/jbc.M100478200 11320086

[B2] Bolos V. Peinado H. Perez-Moreno M. A. Fraga M. F. Esteller M. Cano A. (2003). The transcription factor Slug represses E-cadherin expression and induces epithelial to mesenchymal transitions: a comparison with Snail and E47 repressors. *J. Cell Sci.* 116 499–511. 10.1242/jcs.00224 12508111

[B3] Bottcher R. T. Veelders M. Rombaut P. Faix J. Theodosiou M. Stradal T. E. (2017). Kindlin-2 recruits paxillin and Arp2/3 to promote membrane protrusions during initial cell spreading. *J. Cell Biol.* 216 3785–3798. 10.1083/jcb.201701176 28912124 PMC5674885

[B4] Cabrerizo-Granados D. Pena R. Palacios L. Carillo-Bosch L. Lloreta-Trull J. Comerma L. (2021). Snail1 expression in endothelial cells controls growth, angiogenesis and differentiation of breast tumors. *Theranostics* 11 7671–7684. 10.7150/thno.61881 34335957 PMC8315050

[B5] Cano A. Perez-Moreno M. A. Rodrigo I. Locascio A. Blanco M. J. Del Barrio M. G. (2000). The transcription factor snail controls epithelial-mesenchymal transitions by repressing E-cadherin expression. *Nat. Cell Biol.* 2 76–83. 10.1038/35000025 10655586

[B6] Carpentier G. Berndt S. Ferratge S. Rasband W. Cuendet M. Uzan G. (2020). Angiogensis anlyzer for ImageJ - a comparative morphometric analysis of “endothelial tube formation assay” and “fibrin bead assay”. *Sci. Report* 10:11568. 10.1038/s41598-020-67289-8 32665552 PMC7360583

[B7] Chen W. Epshtein Y. Ni X. Dull R. O. Cress A. E. Garcia J. G. (2015). Role of Integrin beta4 in Lung Endothelial Cell Inflammatory Responses to Mechanical Stress. *Sci. Rep.* 5:16529. 10.1038/srep16529 26572585 PMC4647208

[B8] Chen W. Garcia J. G. Jacobson J. R. (2010). Integrin beta4 attenuates SHP-2 and MAPK signaling and reduces human lung endothelial inflammatory responses. *J. Cell Biochem.* 110 718–724. 10.1002/jcb.22582 20512931 PMC2879705

[B9] Chen W. Sammani S. Mitra S. Ma S. F. Garcia J. G. Jacobson J. R. (2012). Critical role for integrin-beta4 in the attenuation of murine acute lung injury by simvastatin. *Am. J. Physiol. Lung. Cell Mol. Physiol.* 303 L279–L285. 10.1152/ajplung.00361.2011 22683568 PMC3423831

[B10] Comijn J. Berx G. Vermassen P. Verschueren K. Van Grunsven L. Bruyneel E. (2001). The two-handed E box binding zinc finger protein SIP1 downregulates E-cadherin and induces invasion. *Mol. Cell* 7 1267–1278. 10.1016/s1097-2765(01)00260-x 11430829

[B11] Davignon J. (2004). Beneficial cardiovascular pleiotropic effects of statins. *Circulation* 109 III39–III43. 10.1161/01.CIR.0000131517.20177.5a 15198965

[B12] Eastham A. M. Spencer H. Soncin F. Ritson S. Merry C. L. Stern P. L. (2007). Epithelial-mesenchymal transition events during human embryonic stem cell differentiation. *Cancer Res.* 67 11254–11262. 10.1158/0008-5472.CAN-07-2253 18056451

[B13] Eger A. Aigner K. Sonderegger S. Dampier B. Oehler S. Schreiber M. (2005). DeltaEF1 is a transcriptional repressor of E-cadherin and regulates epithelial plasticity in breast cancer cells. *Oncogene* 24 2375–2385. 10.1038/sj.onc.1208429 15674322

[B14] Garcia J. G. Liu F. Verin A. D. Birukova A. Dechert M. A. Gerthoffer W. T. (2001). Sphingosine 1-phosphate promotes endothelial cell barrier integrity by Edg-dependent cytoskeletal rearrangement. *J. Clin. Invest* 108 689–701. 10.1172/JCI12450 11544274 PMC209379

[B15] Hogervorst F. Von Dem Borne A. E. Sonnenberg A. (1990). Cloning and sequence analysis of beta-4 cDNA: an integrin subunit that contains a unique 118 kd cytoplasmic domain. *EMBO J.* 9 765–770. 2311578 10.1002/j.1460-2075.1990.tb08171.x PMC551734

[B16] Hultgren N. W. Fang J. S. Ziegler M. E. Ramirez R. N. Phan D. T. T. Hatch M. M. S. (2020). Slug regulates the Dll4-Notch-VEGFR2 axis to control endothelial cell activation and angiogenesis. *Nat. Comm.* 11:5400. 10.1038/s41467-020-18633-z 33106502 PMC7588439

[B17] Jacobson J. R. (2009). Statins in endothelial signaling and activation. *Antioxid Redox Signal* 11 811–821. 18808324 10.1089/ars.2008.2284 PMC6463996

[B18] Jacobson J. R. Barnard J. W. Grigoryev D. N. Ma S. F. Tuder R. M. Garcia J. G. (2005). Simvastatin attenuates vascular leak and inflammation in murine inflammatory lung injury. *Am. J. Physiol. Lung. Cell Mol. Physiol.* 288 L1026–L1032. 10.1152/ajplung.00354.2004 15665042

[B19] Kelly G. T. Faraj R. Dai Z. Cress A. E. Wang T. (2020). A mutation found in esophageal cancer alters integrin beta4 mRNA splicing. *Biochem. Biophys. Res. Commun.* 529 726–732. 10.1016/j.bbrc.2020.06.078 32736699

[B20] Kurpakus M. A. Quaranta V. Jones J. C. (1991). Surface relocation of alpha 6 beta 4 integrins and assembly of hemidesmosomes in an *in vitro* model of wound healing. *J. Cell Biol.* 115 1737–1750. 10.1083/jcb.115.6.1737 1757471 PMC2289215

[B21] Li G. S. Hou W. Chen G. Yao Y. X. Chen X. Y. Zhang X. G. (2020). Clinical Significance of Integrin Subunit Beta 4 in Head and Neck Squamous Cell Carcinoma. *Cancer Biother. Radiopharm.* 10.1089/cbr.2020.3943 [Epub ahead of print]. 33179959

[B22] Li M. Jiang X. Wang G. Zhai C. Liu Y. Li H. (2019). ITGB4 is a novel prognostic factor in colon cancer. *J. Cancer* 10 5223–5233. 10.7150/jca.29269 31602273 PMC6775604

[B23] Li X. L. Liu L. Li D. D. He Y. P. Guo L. H. Sun L. P. (2017). Integrin beta4 promotes cell invasion and epithelial-mesenchymal transition through the modulation of Slug expression in hepatocellular carcinoma. *Sci Rep* 7:40464. 10.1038/srep40464 28084395 PMC5233967

[B24] Linz-Mcgillem L. A. Moitra J. Garcia J. G. (2004). Cytoskeletal rearrangement and caspase activation in sphingosine 1-phosphate-induced lung capillary tube formation. *Stem Cells Dev.* 13 496–508. 10.1089/scd.2004.13.496 15588507

[B25] Ma J. Van Der Zon G. Goncalves M. Van Dinther M. Thorikay M. Sanchez-Duffhues G. (2021). TGF-beta-Induced Endothelial to Mesenchymal Transition Is Determined by a Balance Between SNAIL and ID Factors. *Front. Cell Dev. Biol.* 9:616610. 10.3389/fcell.2021.616610 33644053 PMC7907445

[B26] Masugi Y. Yamazaki K. Emoto K. Effendi K. Tsujikawa H. Kitago M. (2015). Upregulation of integrin beta4 promotes epithelial-mesenchymal transition and is a novel prognostic marker in pancreatic ductal adenocarcinoma. *Lab. Invest* 95 308–319. 10.1038/labinvest.2014.166 25599535

[B27] Masunaga T. Niizeki H. Yasuda F. Yoshida K. Amagai M. Ishiko A. (2015). Splicing abnormality of integrin beta4 gene (ITGB4) due to nucleotide substitutions far from splice site underlies pyloric atresia-junctional epidermolysis bullosa syndrome. *J. Dermatol. Sci.* 78 61–66. 10.1016/j.jdermsci.2015.01.016 25728941

[B28] Meng X. Liu P. Wu Y. Liu X. Huang Y. Yu B. (2020). Integrin beta 4 (ITGB4) and its tyrosine-1510 phosphorylation promote pancreatic tumorigenesis and regulate the MEK1-ERK1/2 signaling pathway. *Bosn. J. Basic Med. Sci.* 20 106–116. 10.17305/bjbms.2019.4255 31242404 PMC7029197

[B29] Mitra S. Epshtein Y. Sammani S. Quijada H. Chen W. Bandela M. (2021). UCHL1, a deubiquitinating enzyme, regulates lung endothelial cell permeability *in vitro* and *in vivo*. *Am. J. Physiol. Lung. Cell Mol. Physiol.* 320 L497–L507. 10.1152/ajplung.00492.2020 33438509 PMC8238159

[B30] Nagata M. Noman A. A. Suzuki K. Kurita H. Ohnishi M. Ohyama T. (2013). ITGA3 and ITGB4 expression biomarkers estimate the risks of locoregional and hematogenous dissemination of oral squamous cell carcinoma. *BMC Cancer* 13:410. 10.1186/1471-2407-13-410 24006899 PMC3844399

[B31] Nikolopoulos S. N. Blaikie P. Yoshioka T. Guo W. Puri C. Tacchetti C. (2005). Targeted deletion of the integrin beta4 signaling domain suppresses laminin-5-dependent nuclear entry of mitogen-activated protein kinases and NF-kappaB, causing defects in epidermal growth and migration. *Mol. Cell Biol.* 25 6090–6102. 10.1128/MCB.25.14.6090-6102.2005 15988021 PMC1168825

[B32] Pal A. Barrett T. F. Paolini R. Parikh A. Puram S. V. (2021). Partial EMT in head and neck cancer biology: a spectrum instead of a switch. *Oncogene* 40 5049–5065. 10.1038/s41388-021-01868-5 34239045 PMC8934590

[B33] Rognoni E. Ruppert R. Fassler R. (2016). The kindlin family: functions, signaling properties and implications for human disease. *J. Cell Sci.* 129 17–27. 10.1242/jcs.161190 26729028

[B34] Rong F. Li Y. Jiang N. Ren B. Zang C. Liu L. (2020). Inactivation of endothelial ZEB1 impedes tumor progression and sesitizes tumors to conventional therapies. *J. Clin. Invest* 130 1252–1270. 10.1172/JCI131507 32039918 PMC7269596

[B35] Stepp M. A. Spurr-Michaud S. Tisdale A. Elwell J. Gipson I. K. (1990). Alpha 6 beta 4 integrin heterodimer is a component of hemidesmosomes. *Proc. Natl. Acad. Sci. U.S.A.* 87 8970–8974. 10.1073/pnas.87.22.8970 2247472 PMC55082

[B36] Suarez-Arnedo A. Torres Figuroa F. Clavijo C. Arbelaez P. Cruz J. C. Munoz-Camargo C. (2020). An image J plugin for the high throughput image analysis of *in vitro* scratch wound healing assays. *PLoS One* 15:e0232565. 10.1371/journal.pone.0232565 32722676 PMC7386569

[B37] Thiery J. P. (2002). Epithelial-mesenchymal transitions in tumour progression. *Nat. Rev. Cancer* 2 442–454. 12189386 10.1038/nrc822

[B38] Voon D. C. Huang R. Y. Jackson R. A. Thiery J. P. (2017). The EMT spectrum and therapeutic opportunities. *Mol. Oncol.* 11 878–891. 10.1002/1878-0261.12082 28544151 PMC5496500

[B39] Xu-Dubois Y. C. Peltier J. Brocheriou I. Suberbielle-Boissel C. Djamali A. Reese S. (2016). Markers of Endothelial-to-Mesenchymal Transition: Evidence for Antibody-Endothelium Interaction during Antibody-Mediated Rejection in Kidney Recipients. *J. Am. Soc. Nephrol.* 27 324–332. 10.1681/ASN.2014070679 25995444 PMC4696562

[B40] Zeisberg E. M. Tarnavski O. Zeisberg M. Dorfman A. L. Mcmullen J. R. Gustafsson E. (2007). Endothelial-to-mesenchymal transition contributes to cardiac fibrosis. *Nat. Med.* 13 952–961. 17660828 10.1038/nm1613

[B41] Zhang Q. Dong J. Yu Z. (2020). Pleiotropic use of Statins as non-lipid-lowering drugs. *Int. J. Biol. Sci.* 16 2704–2711. 10.7150/ijbs.42965 33110390 PMC7586431

